# Supplementary material for: Unprofessional peer reviews disproportionately harm underrepresented groups in STEM
Source: PeerJ. 2019 Dec 12;7:e8247. doi: 10.7717/peerj.8247 (PMC6911688; doi:10.7717/peerj.8247)
Supplement: Supplemental Information 1 [file peerj-07-8247-s001.pdf]

**Unprofessional peer reviews disproportionately harm underrepresented groups in STEM**

Nyssa J Silbiger[1*] and Amber D Stubler[2*]

**Supplemental Tables and Figures**

**Table S1: Breakdown of raw data for each gender for each of the three models: a) scientific aptitude, b) productivity, and c) career advancement.** Values are the raw counts for each category (1-5).

| | **1** | **2** | **3** | **4** | **5** | **Grand Total** |
|---|---|---|---|---|---|---|
| Scientific Aptitude | | | | | | |
| Men | 97 | 59 | 34 | 33 | 24 | **247** |
| Non-binary | 1 | 1 | 1 | 1 | 0 | **4** |
| Women | 73 | 63 | 95 | 80 | 55 | **366** |
| **Grand Total** | **171** | **123** | **130** | **114** | **79** | **617** |
| Productivity | | | | | | |
| Men | 89 | 54 | 46 | 35 | 25 | **249** |
| Non-binary | 0 | 0 | 0 | 3 | 1 | **4** |
| Women | 63 | 79 | 110 | 70 | 45 | **367** |
| **Grand Total** | **152** | **133** | **156** | **108** | **71** | **620** |
| Career Advancement | | | | | | |
| Men | 124 | 40 | 43 | 22 | 19 | **248** |
| Non-binary | 2 | 0 | 1 | 0 | 1 | **4** |
| Women | 124 | 91 | 63 | 47 | 41 | **366** |
| **Grand Total** | **250** | **131** | **107** | **69** | **61** | **618** |

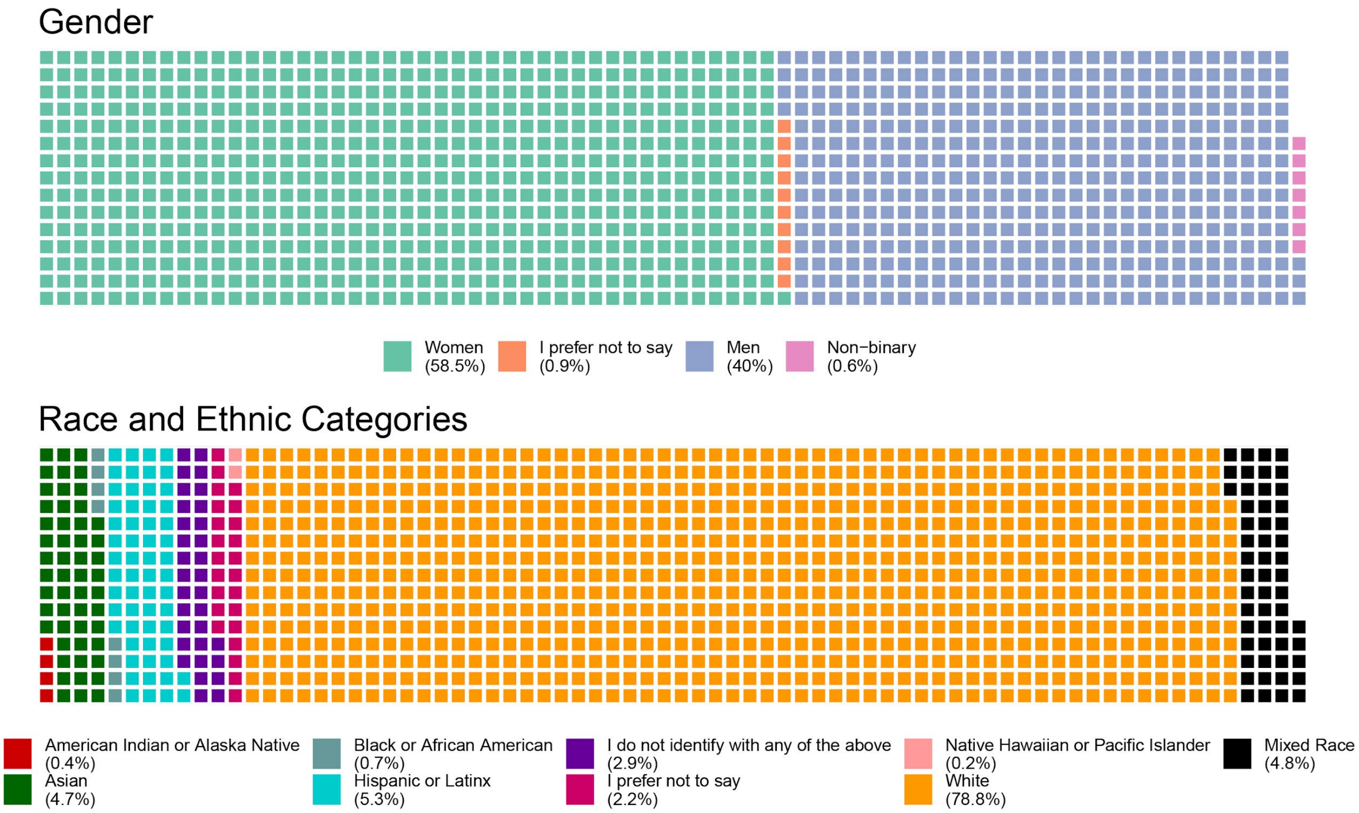


**Figure S1: Percentage of all respondents by gender and race/ethnicity for all categories in the survey.**

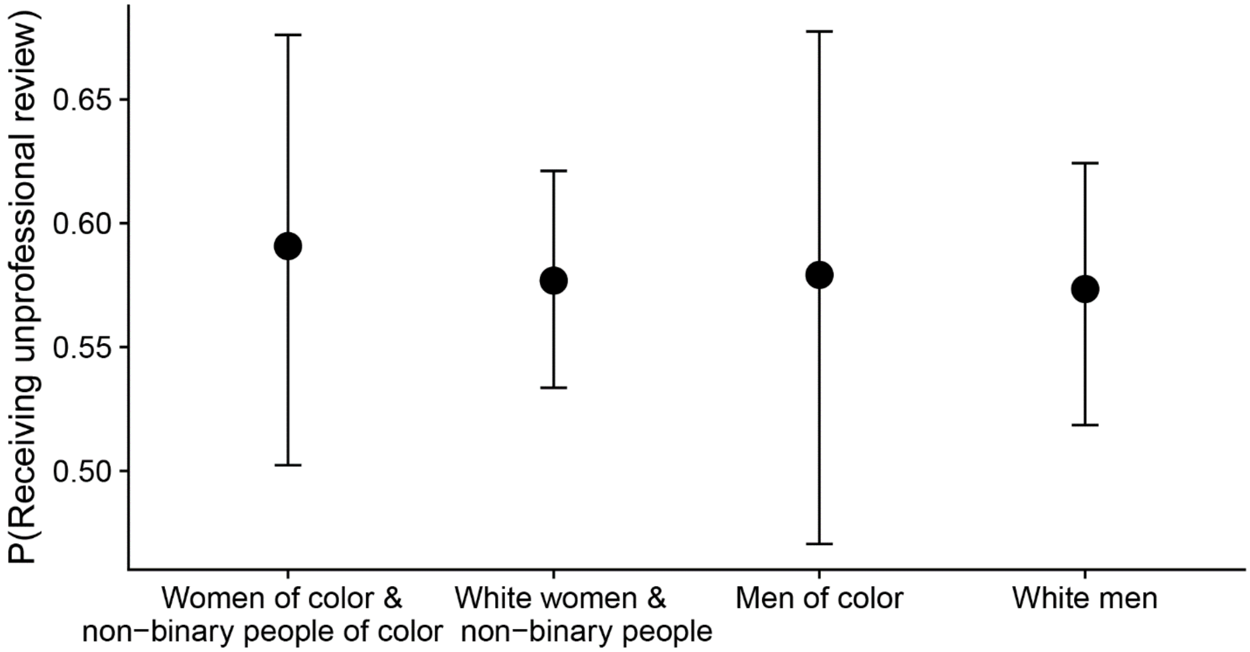


**Figure S2: Results from Bayesian logistic regression showing the probability of receiving an unprofessional peer review by intersectional groups.** Symbols are medians ± 95% BCI. There is no significant difference in the probability of receiving an unprofessional review among these four groups.
