# Supplementary material for: Unprofessional peer reviews disproportionately harm underrepresented groups in STEM
Source: PeerJ. 2019 Dec 12;7:e8247. doi: 10.7717/peerj.8247 (PMC6911688; doi:10.7717/peerj.8247)
Supplement: Supplemental Information 2 — Detailed information on survey distribution for this study. [file peerj-07-8247-s002.docx]

Distribution Methods

Stubler and Silbiger sent **direct personalized emails** to chairs of departments at multiple historically black colleges and universities, minority serving institutions, state schools, and private academic institutions (n=20) with the request to forward to any interested colleagues at their institution or beyond. In addition, emails were also sent to colleagues at various institutions (both academic and non-academic) with the request to pass along the survey to relevant parties. An example of the base email is included below (note that this email was personalized to the individual/organization).

Silbiger and Stubler **posted to several STEM field listservs** to advertise the survey (content of posts were similar to the email). Note that while we requested the survey to be posted on the listservs below, we did not subscribe to each of the listservs to check whether it was successfully distributed.

Ecolog – Ecological Society of America listserv

Coral-list – NOAA-run listserv for anyone involved in coral reef conservation and research

MarMam – Researchers and managers working with marine mammals

Algae-L – Phycology, algae from terrestrial, freshwater or marine environments

Fish Ecolog-L – Ichthyology, empirical and theoretical research and assessment issues related to the ecology of fish and fisheries

Biogeosciences list – Biogeosciences/geology

Ocean Science list – Oceanography, marine science

EvolDir – evolutionary biologists, population biologists and related areas

Marine-B – MArine Research Information NEtwork on Biodiversity, is for communication related to marine biodiversity research

Isogeochem – Anyone with an interest in stable isotope geochemistry

Earth sciences – environmental sciences

Env-Chem – environmental chemistry

PPEM list – physical properties of earth materials

COGDEVSOC – Cognitive Development Society listserv

Stubler and Silbiger contacted the following list of **societies and organizations** with the request to distribute or forward the survey to members. It is unknown whether all of these organizations passed along the information.

Coastal and Estuarine Research Federation (society email listserv; <https://www.cerf.science/>)

Benthic Ecology Meeting (society email listserv; <https://www.bemsociety.org/>)

Society for Advancing Chicanos/Hispanics and Native Americans in Science (SACNAS; society email listserv, <https://www.sacnas.org/>)

Latino STEM alliance (<https://www.latinostem.org/>)

Latinas in STEM (<http://www.latinasinstem.com/>)

Latinos in science and engineering (<https://mymaes.org/>)

Latino STEM Association (<http://www.latinostem.net/>)

500 Women Scientists (<https://500womenscientists.org/>)

Ford Fellows (<https://sites.nationalacademies.org/pga/fordfellowships/>)

Both Stubler and Silbiger **advertised the survey on social media** using on our own personal and professional Twitter accounts; these posts were re-tweeted widely, but there is no way to discern how many individuals interacted with the survey using this platform. Silbiger posted the survey link on the Nancy Foster Scholarship (NOAA) Facebook group.

In addition to the groups we contacted directly, we **received emails from several people interested in disseminating our study** further and we are aware of the following groups who received notification of our survey through second party distributors: Peer Community In, Porifera Listserv, NOAA Fisheries Department.

**Form email sent directly to individuals from either Silbiger or Stubler**

Good morning/afternoon,

Dr. Amber Stubler at Occidental College and Dr. Nyssa Silbiger at California State University, Northridge are conducting an **anonymous** research study to better understand the impacts of receiving unprofessional peer reviews in STEM fields, the frequency at which they are received, and the subsequent impacts on a researcher’s perception of their abilities.

If you have ever published a peer-reviewed study as first author, please consider filling out this anonymous survey ([https://oxy.qualtrics.com/jfe/form/SV_2h5lUZkxR28deZv](https://urldefense.proofpoint.com/v2/url?u=https-3A__oxy.qualtrics.com_jfe_form_SV-5F2h5lUZkxR28deZv&d=DwMFaQ&c=Oo8bPJf7k7r_cPTz1JF7vEiFxvFRfQtp-j14fFwh71U&r=N_8vOX_1dSpuAq1PlVfBE95Ane8sbu_AGy7K1PhBMs4&m=eODxBpevChFDuRsmKOFC-1kqIBGJ7GWCpo8f5Rf3fII&s=DRHQVEeEuY6f3JoyzgiVN-BE6zvBnSjjEOePITnFI18&e=)**)***.* The survey will take **2-10 minutes to complete**. Information collected from this study will be kept confidential and no unique personal identifying information will be collected.

Please consider distributing widely among students and STEM professionals (both academic and non-academic).

If you have any questions or concerns about the research, you can contact Dr. Amber Stubler at [astubler@oxy.edu](mailto:astubler@oxy.edu) or Dr. Nyssa Silbiger at [nyssa.silbiger@csun.edu](mailto:nyssa.silbiger@csun.edu).

<https://oxy.qualtrics.com/jfe/form/SV_2h5lUZkxR28deZv>

Thank you very much for your time.

Dr. Nyssa Silbiger and Dr. Amber Stubler

(Occidental College #IRB00009103, California State University, Northridge #IRB00001788)
