# Supplementary material for: Unprofessional peer reviews disproportionately harm underrepresented groups in STEM
Source: PeerJ. 2019 Dec 12;7:e8247. doi: 10.7717/peerj.8247 (PMC6911688; doi:10.7717/peerj.8247)
Supplement: Supplemental Information 3 [file peerj-07-8247-s003.pdf]

Informed Consent Statement

Title of study: ***Understanding the impacts of unprofessional peer reviews on early career scientists.***

Please read the following statement of consent for participation in this research study.

You are invited to participate in an **anonymous** research study conducted by Dr. Amber Stubler, a faculty member from the Biology Department at Occidental College, and Dr. Nyssa Silbiger, a faculty member from the Biology Department at California State University, Northridge. You must be at least 18 years of age to participate in this study, **information collected from this study will be kept confidential and no unique personal identifying information will be collected**.

The purpose of this study is to understand the impacts of receiving unprofessional peer review—which we have defined as any comment that is unfair, unethical, or irrelevant to the nature of the work; this includes comments that:
1) lack constructive criticism,
2) are directed at the author, rather than the nature or quality of the work,
3) use personal opinions of the author/work rather than evidence-based criticism, or
4) are "mean-spirited" or cruel.

Specifically, we aim to understand the nature of the unprofessional peer reviews, the frequency at which they are received, and the subsequent impacts on a researcher's perception of their abilities. If you agree to take part in this anonymous study, you will be asked to complete an online survey that asks

specific questions about your educational training and background, the number of peer-reviewed publications you've submitted, the nature of the peer-reviews received, and any perceived or realized impacts that have resulted from unprofessional peer reviews. The survey will take 2-15 minutes to complete, with the option to include specific examples of unprofessional peer-reviews received.

Your decision to participate or decline participation in this study is voluntary and you may skip any questions that you do not want to answer by selecting 'I prefer not to say'. You are free to stop taking the survey at any time without penalty, and with no loss of benefits to which you were otherwise entitled.

There are no anticipated risks or discomforts to your participation in this study beyond those encountered in daily life. However, remembering an unpleasant experience may cause you some emotional discomfort and you may wish to discuss this with a friend, family member, or health care professional if needed. To the best of our ability your answers in this study will remain confidential as stated below.

Although you may not directly benefit from this research, by participating in this study you will help to further our understanding of the inherent biases that are associated with the single-blind peer-review system for scientific articles/publications.

Again, information collected from this study will be kept confidential and no unique personal identifying information will be collected. Only the two principal investigators will have access to the complete anonymous responses; all presented data will be grouped and analyzed by Drs. Silbiger and/or Stubler, with the exception of specific examples of unprofessional peer reviews. Digital data will be stored in password protected computer files. No names of participants surveyed will be collected in this study. Data collected from this study will be analyzed and presented at meetings or conferences and/or published by Drs. Silbiger and Stubler. Data derived from this study may be made publicly available after publication, following the journal's data reporting standards; however, **no identifying information will be included**.

You will not be paid for participating in this study.

If you have any questions or concerns about the research, you can contact Dr. Amber Stubler at astubler@oxy.edu or Dr. Nyssa Silbiger at nyssa.silbiger@csun.edu. If you have any questions or concerns regarding your rights as a subject in this study, you may contact the Institutional Review Board Office at Occidental College in Los Angeles, CA, 90041 at hsrrc@oxy.edu or (323) 259-2921.

Please print a copy of this page for your records.

By clicking "I agree" below, you are indicating that you are at least 18 years of age, have read and understood this consent form, and voluntarily agree to participate in this **anonymous** research study.

- I agree
- I do not agree

Demographics (everyone answers)

Please choose the gender that you currently identify as:

- Female
- Male
- Non-binary
- I prefer not to say

Please identify the highest level of education completed (as of today):

- Bachelor's degree
- Master's degree
- Doctoral degree
- Other professional degree
- I prefer not to say

Choose the career stage that best describes your current situation:

- Graduate student working toward Master's degree
- Graduate student working toward Ph.D. or other doctoral degree
- Post-doctoral researcher
- Assistant Professor
- Associate Professor
- Full Professor
- Non-tenure track full-time instructor
- Non-tenure track part-time instructor or Adjunct
- State or Federal employee at non-degree granting institution or agency
- Private Industry/Research Facility
- I prefer not to say
- None of the above

Do you currently reside in the United States?

- Yes
- No

- ○ I prefer not to say

In which country do you currently reside?

[▾]

If you are currently a professional scientist (non-student), which of the following best describes your place of employment?

- ○ Institution of higher learning
- ○ State or Federal research agency
- ○ Industry/Privately-owned company
- ○ Other
- ○ I am currently a student and/or does not apply
- ○ I prefer not to say

How would you best describe your **broad** field of research expertise? Select all that apply:

- ☐ Biology
- ☐ Chemistry
- ☐ Ecology/Evolution
- ☐ Geology/Earth Sciences
- ☐ Oceanography
- ☐ Physics

- ☐ Mathematics
- ☐ Computer Science
- ☐ Engineering
- ☐ Psychology
- ☐ Biomedical
- ☐ Astronomy/Space sciences
- ☐ Other
- ☐ I prefer not to say
- ☐ Social Science

What racial and/or ethnic categories do you identify as? Select all that apply.

- ☐ American Indian or Alaska Native
- ☐ Asian
- ☐ Black or African American
- ☐ Hispanic or Latinx
- ☐ Native Hawaiian or Pacific Islander
- ☐ White
- ☐ I do not identify with any of the above
- ☐ I prefer not to say

Do you identify as a first generation student (the first person in your immediate family to earn a Bachelor's degree)?

- ○ Yes

- No
- Not sure
- I prefer not to say

Is English your primary language?

- Yes
- No
- I prefer not to say

Yes/No Question about Peer Reviews

For the purpose of this survey, an **unprofessional peer review** is defined as any comment that is unethical, or irrelevant to the nature of the work; this includes comments that 1) lack constructive criticism in an unfair way, 2) are directed at the author rather than the nature or quality of the work, 3) invoke personal opinions of the author/work rather than evidence-based criticism, or 4) are "mean-spirited" or cruel.

Specific examples of unprofessional peer reviews:
Lacks constructive criticism*:**
"First of all, it would be nice to know something about the fundamental way in which [this phenomenon] will work, which can partly be adduced by looking at-LOOKING AT-the critters.

Directed at author, rather than work:
"the authors do not appear to be overly familiar with either ocean acidification, water chemistry, [organism] physiologies or [subject matter of paper]"

“In short, this piece of research bears all the hallmarks of some bright people who saw an opportunity in a currently hot field of research, and thought they would jump in because, after all, how hard could it be? I have scanned the resumes of every one of the authors, and have come to the conclusion that they are indeed very bright people who could have used some good advice before starting this. The passage of this manuscript would have been much easier, and I would not have had to work so hard"

Reflects personal opinions rather than evidence-based criticism:
"they can forget [Dr. X’s] work, but [Dr. Y’s] work is rock-solid"

“Mean-spirited” or cruel:
“The phrases I have so far avoided using in this review are, ‘lipstick on a pig’, and ‘bullshit baffles brains’.”

*******We recognize that there are professional and relevant reviewer statements that may lack constructive criticism; however, we are specifically focusing on those statements that are *unprofessional* in nature.

Have you ever received an unprofessional peer-review, as defined above, as the lead author?

- ○ Yes
- ○ No
- ○ I prefer not to say

Yes Path

According to the definition used by this survey, which of the following characterizes the unprofessional peer review(s), select all that apply:

- [ ] Lacked constructive criticism
- [ ] Directed at author rather than work
- [ ] Reflected personal opinions rather than evidence-based criticism
- [ ] Mean-spirited or cruel

At what point in your career stage have you received an unprofessional peer review. Select all that apply.

- [ ] Undergraduate student
- [ ] Master's student
- [ ] Doctoral student
- [ ] Post-doctoral researcher/Independent scientist
- [ ] Industry professional
- [ ] Assistant professor
- [ ] Associate professor
- [ ] Full professor
- [ ] Non-tenure track academic scientist
- [ ] I prefer not to say

How many times have you received an unprofessional peer review as the lead author (from independent submissions)?

To date, how many accepted, peer-reviewed papers have you been the lead author of?

When you **began** your graduate education/training, was your goal to become an academic scientist?

- Yes
- No
- Unsure
- I prefer not to say

Is this still your goal and/or have you achieved this goal:

- Yes
- No
- Unsure
- I prefer not to say

Did receiving unprofessional peer reviews contribute to your decision to shift career path away from academia?

- Yes, completely
- Yes, partially
- No
- I prefer not to say

After receiving your **first** unprofessional review, did you:

- Revise and resubmit (to any journal)
- Did not revise or resubmit the manuscript to any journal
- I do not recall or prefer not to say

How long did it take you to revise and resubmit?

- 0-6 months
- 6-12 months
- 12-24 months
- more than 24 months
- I prefer not to say

Was your decision not to revise and resubmit directly related to the unprofessional nature of the review?

- Yes, partially
- Yes, completely
- No
- I prefer not to say

After receiving your first unprofessional review, did you have a mentor/advisor or support system that influenced the above decision?

- Yes
- No

To what degree did the unprofessional peer review(s) make you doubt your **scientific aptitude**?
(1-5) 1 = not at all, 5 = I fully doubted my scientific abilities.

To what degree do you feel that receiving the unprofessional review(s) limited your **overall productivity**?
Please rate this from 1-5. 1 = not at all, 5 = greatly limited number of publications per year

To what degree do you feel that receiving the unprofessional review(s) delayed **career advancement**?
Please rate this from 1-5. 1 = not at all, 5 = greatly limited or delayed career advancement

Were any of the unprofessional peer reviews received publicly visible alongside the paper once published (examples of journals that do this: PeerJ, Journal of Marine Science)?

- Yes
- No
- I prefer not to say

If you are willing, please include any example(s) of unprofessional peer reviews that you've received. Please include exact quotes only (do not paraphrase); the only exception is that you may choose to remove any personal or identifying information such as field of study, pronouns, etc. You may choose not to contribute for any reason; if this is the case, please leave blank.

Example: *"the authors do not appear to be overly familiar with either [this phenomenon], water chemistry, physiologies [of the organisms] or [subject matter of paper]"*

As a reminder, this survey is completely anonymous and has collected no personal identifying information. Do you give permission for this quote to be shared/distributed? Please check your selection:

- Yes, you may use the exact quote as entered above.
- No, please do not share my example.
- I prefer not to contribute.

If you have any additional comments, please add them below. Please do not include any identifying information that will compromise the anonymity of your response.

Submit certification

By clicking the submit button, you certify that all of the above information is true and accurate, to the best of your abilities, and you have shared this information willingly.

○ Submit

No Path

To date, how many accepted, peer-reviewed papers have you been lead author of?

[ ]

When you **began** your graduate education/training, was your goal to become an academic scientist?

○ Yes
○ No
○ Unsure
○ I prefer not to say

Is this still your goal/have you achieved this goal:

○ Yes
○ No
○ Unsure
○ I prefer not to say

If you have any additional comments, please add them below. Please do not include any identifying information that will compromise the anonymity of your response.

Powered by Qualtrics
